# Diagnostic instruments for the assessment of disruptive mood dysregulation disorder: a systematic review of the literature

**DOI:** 10.1007/s00787-021-01840-4

**Published:** 2021-07-07

**Authors:** Ines Mürner-Lavanchy, Michael Kaess, Julian Koenig

**Affiliations:** 1 University Hospital of Child and Adolescent Psychiatry and Psychotherapy, University of Bern, Bolligenstrasse 111, 3000 Bern 60, Switzerland; 2 Section for Translational Child and Adolescent Psychiatry, Department of Child and Adolescent Psychiatry, Centre for Psychosocial Medicine, University of Heidelberg, Heidelberg, Germany; 3 Section for Experimental Child and Adolescent Psychiatry, Department of Child and Adolescent Psychiatry, Centre for Psychosocial Medicine, University of Heidelberg, Heidelberg, Germany

**Keywords:** Disruptive mood dysregulation disorder, Irritability, Diagnostics, Measurement, Systematic review of the literature

## Abstract

Disruptive mood dysregulation disorder (DMDD) involves non-episodic irritability and frequent severe temper outbursts in children. Since the inclusion of the diagnosis in the DSM-5, there is no established gold-standard in the assessment of DMDD. In this systematic review of the literature, we provide a synopsis of existing diagnostic instruments for DMDD. Bibliographic databases were searched for any studies assessing DMDD. The systematic search of the literature yielded *K* = 1167 hits, of which *n* = 110 studies were included. The most frequently used measure was the Kiddie Schedule for Affective Disorders and Schizophrenia DMDD module (25%). Other studies derived diagnostic criteria from interviews not specifically designed to measure DMDD (47%), chart review (7%), clinical diagnosis without any specific instrument (6%) or did not provide information about the assessment (9%). Three structured interviews designed to diagnose DMDD were used in six studies (6%). Interrater reliability was reported in 36% of studies (ranging from *κ* = 0.6–1) while other psychometric properties were rarely reported. This systematic review points to a variety of existing diagnostic measures for DMDD with good reliability. Consistent reporting of psychometric properties of recently developed DMDD interviews, as well as their further refinement, may help to ascertain the validity of the diagnosis.

**Supplementary Information:**

The online version contains supplementary material available at 10.1007/s00787-021-01840-4.

## Introduction

Disruptive mood dysregulation disorder (DMDD) is a relatively new diagnosis, which has been introduced to the domain of depressive disorders in the fifth version of the Diagnostic and Statistical Manual of Mental Disorders (DSM-5) in 2013 [1]. The diagnosis was endorsed by DSM-5 work groups to address concerns that children with pathological irritability and temper outbursts/anger were being inappropriately diagnosed with bipolar disorder [2]. The diagnosis of bipolar disorder did not accurately capture the non-episodic nature of those children’s symptoms and therefore, might have led to questionable treatment decisions [3]. The development of the DMDD diagnosis was based on the description of a broad phenotype of pediatric bipolar disorder called severe mood dysregulation (SMD) by Leibenluft and colleagues in 2003 [4]. In addition to irritability and anger, the latter required symptoms of chronic hyperarousal (e.g. agitation, distractibility, racing thoughts, insomnia, pressured speech or intrusiveness). Increasing evidence of the clinical distinction between episodic and non-episodic irritability and anger as well as distinct pathophysiology finally led to the formulation of the new diagnosis [2, 5–7].

DMDD involves non-episodic anger or irritability and frequent severe temper outbursts over a period of at least one year in pediatric patients aged 6–18 years [1]. Temper outbursts occur on average three or more times per week, can occur verbally or behaviorally (e.g. physical aggression towards objects or persons), their duration or intensity is inappropriate to the situation and they are inconsistent with the child’s developmental level. DMDD is characterized by persistent irritable and angry mood between temper outbursts in at least two of three settings (i.e. at home, at school, with peers). While the average age of onset is suggested to be 5 years of age [2], the diagnosis is assigned from age 6, as the identification of pathology before this age is difficult due to normal variations in preschool behavior [8].

The prevalence of DMDD ranges from 0.8% to 3.3%, with 2–3% in preschool children, 1–3% in 9–12 year-olds, and 0–0.12% in adolescents [9–11]. Although the prevalence of DMDD decreases with increasing age, individuals with a history of DMDD are at higher risk for adult depression and anxiety, adverse health outcomes, low educational attainment, poverty, and reported police contact, compared to healthy and clinical controls with other psychiatric conditions [11]. Prevalence estimates differ between studies because there is substantial diagnostic variability in the adherence to DSM-5 criteria with respect to the frequency of outbursts, the duration of irritability or the exclusion criteria.

Comorbidity is one of the obstacles which have been reported around the DMDD diagnosis [12]. The majority of patients with DMDD have at least one other comorbid psychiatric disorder, of which oppositional defiant disorder (ODD) or depressive disorders are most commonly reported [10]. In addition, there is substantial diagnostic overlap with childhood psychiatric disorders such as ODD, intermittent explosive disorder or attention deficit hyperactivity disorder (ADHD), questioning the validity of the diagnosis as a distinct disorder [13–15]. Correspondingly, in the International Classification of Diseases and Related Health Problems (ICD‐11), DMDD will be listed as a subtype “with chronic irritability‐anger” of oppositional defiant disorder [16].

The diagnostic challenges may, at least in part, be due to difficulties in its assessment [17]. As such, symptoms of DMDD are not unique to children referred for psychiatric services. Hence, many existing measures provide questions which assess symptoms relevant to DMDD (e.g. irritability is measured but considered a nonspecific indicator and is related to several other psychiatric disorders) [12]. Moreover, structured interviews or questionnaires specifically developed to diagnose DMDD are still in their infancy. Consequently, there is currently no gold standard or broad consensus regarding the clinical assessment of DMDD.

In this systematic review of the literature, we aimed to provide a synopsis of all measures that have been used in diagnosing DMDD since the advent of the diagnosis in 2013. Study characteristics of the included studies, quantities of used diagnostic measures, and psychometric properties, where applicable, are reported and discussed. The results of this systematic review of the literature might guide future research in the selection of appropriate tools to diagnose DMDD in the clinical and research setting.

## Methods

This systematic review was conducted in accordance with the Preferred Reporting Items for Systematic Review and Meta-Analyses (PRISMA) checklist [18]. The protocol was pre-registered in the International Prospective Register of Systematic Reviews (PROSPERO) and may be accessed under the registration number CRD 42020165496.

### Literature search

The goal of the literature search was to identify any studies assessing DMDD. Therefore, a broad search strategy was formulated. The full electronic search strategy of the systematic literature search in the PubMed database (https://pubmed.ncbi.nlm.nih.gov) was: ("Disruptive Mood Dysregulation*") OR ("DMDD"). No limits or filters were added to this search. PubMed, Embase, PsycINFO, and Web of Science databases were scrutinized for relevant literature published from 2013 to 31st March 2020. We used identical search terms in all databases. Further, reference lists of publications identified through database search were screened for potentially pertinent studies not identified in the initial search. To reflect the broadest use of tools to diagnose DMDD, in research as well as in the clinic, we included any regular article, case report, or conference abstract published in any of the searched databases.

### Study selection

Studies were excluded if they (a) did not include patients with diagnosed DMDD; or (b) a full text was not available. Prior to a full-text review, the titles, abstracts, and methods sections of the articles identified through database searches were screened for the eligibility criteria outlined above by two independent reviewers until consensus was reached.

### Data extraction

A digital data extraction sheet was developed and refined during the data extraction process. The following data were extracted if available: general information and identifying features of the study, i.e., full reference, year of publication, and country of study origin. Additionally, the article type was identified, comprising regular articles, conference abstracts, or case reports. All article types were included to cover the full breadth of tools available for research and clinical purposes. Magnitudes and percentages of all outcome variables were given for all study types included as well as for abstracts only. Further data extracted comprised details on the study design, study population, sample size, and age range. The main outcome was the tool used to diagnose DMDD, including the rater (clinician, parent, self) and whether psychometric properties had been assessed. Where possible, information about the number of items, administration time, and availability of the tool (licensed vs. free of cost) in different languages was obtained. Authors were contacted to provide details if any of the information of interest was not provided in the study.

## Results

### Search results

**Fig. 1:**
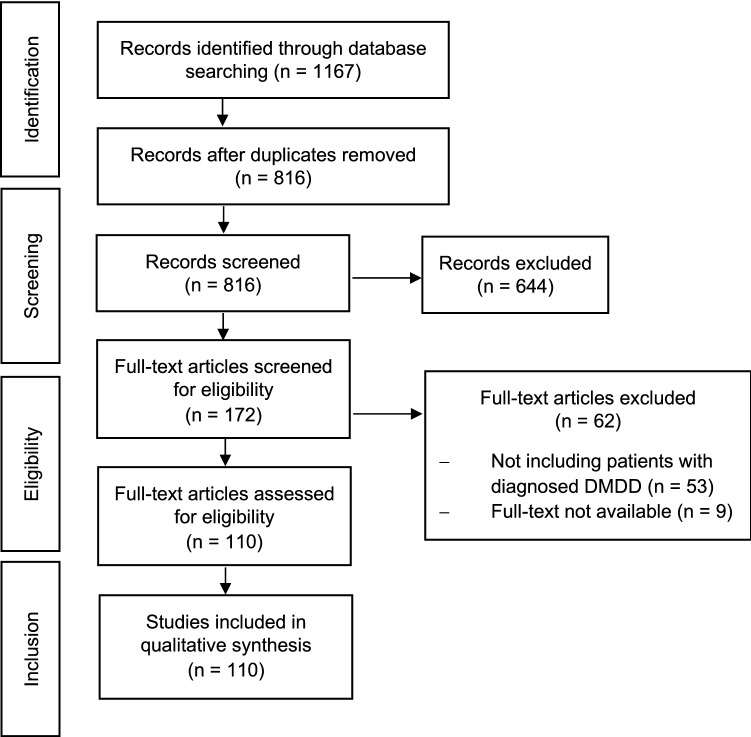
PRISMA flow diagram depicting the study selection process

The first literature search, conducted on January 22, 2020, yielded *K* = 1149 records (PubMed *k* = 168, PsycINFO *k* = 471, Web of Science *k* = 201, Embase *k* = 309). Search updates identical to the first search were carried out on May 26, 2020, yielding an additional *k* = 18 records. *K* = 351 duplicates were removed from the *K* = 1167 records screened for eligibility. Of the *k* = 172 full-text articles screened for eligibility, a further *k* = 53 studies were excluded as they did not include patients with diagnosed DMDD and *k* = 9 because a full text was not obtainable*.* The PRISMA flow diagram of the full process of study selection is depicted in Fig. 1.

### Included studies

From the initial base of records, *k* = 110 studies fulfilled all inclusion criteria and were retained for qualitative syntheses.

General study characteristics of the included studies are described in Table 1. Of the included studies, *k* = 58 were regular articles (52.7%), while there were *k* = 41 conference abstracts (37.3%) and *k* = 11 case reports (10.0%). Most of the studies included a clinical sample (*k* = 83, 75.5%; *k* = 36 abstracts, 32.7%), some were population-based (*k* = 12, 10.9%; *k* = 2 abstracts, 1.8%), case studies (*k* = 11, 10.0%; *k* = 0 abstracts), cohort studies (*k* = 3, 2.7%; *k* = 1 abstract, 0.9%) and *k* = 1 study was among youth in the juvenile justice setting (0.9%; *k* = 0 abstracts). *K* = 85 studies included unique samples (77.3%; *k* = 30 abstracts), while *k* = 25 articles (22.7%; *k* = 11 abstracts) reported data from overlapping samples (see Table 1 for details). *K* = 86 studies were conducted prospectively (78.2%; *k* = 35 abstracts, 31.8%) and *k* = 24 retrospectively (21.8%; *k* = 7 abstracts, 6.4%). Among the prospective studies, *k* = 7 assessed DMDD retrospectively (6.4%; *k* = 1 abstracts, 0.9%).

**Table 1:** Study characteristics by year of publication

Authors	Year	Country of origin	Article type	Study type	Study design	Sample	*N* (% female)	Age (range)^5^
Copeland et al. [10]	2013	USA	Regular article	Population-based	Prospective	Community	3258 (50)	2–17
Copeland et al. [11]	2014	USA	Regular article	Population-based	Prospective^2^	Population	1420 (47)	10–25
Dougherty et al. [54]	2014	USA	Regular article	Population-based	Prospective	Community	462 (46)	6
Parmar et al. [55]	2014	USA	Case report	Case study	Retrospective	Outpatients	1 (0)	15
Roy et al. [43]	2014	USA	Case report	Case study	Retrospective	Outpatients	1 (0)	8
Sparks et al. [56]	2014	USA	Regular article	Clinical	Prospective^2^	Outpatients and community controls	616 (NA)	6–17
Deveney et al. [57]	2015	USA	Regular article	Clinical	Prospective^2^	Outpatients	194 (35)	7–17
Estrada Prat et al. [58]	2015	Spain	Conference abstract	Clinical	Prospective	Outpatients	8 (25)	7–18
Mitchell et al.^a^ [59]	2015	Canada	Conference abstract	Clinical	Prospective	Outpatients	116 (NA)	NA
Schilpzand et al.^b^ [60]	2015	Australia	Conference abstract	Clinical	Prospective	patients	179 (31)	6–8
Stoddard et al. [61]	2015	USA	Conference abstract	Clinical	Prospective	patients and healthy controls	89 (48)^4^	8–18
Tseng et al. [62]	2015	USA	Conference abstract	Clinical	Prospective	patients and healthy controls	75 (53)	8–18
Uran et al.^c^ [63]	2015	Turkey	Regular article	Clinical	Prospective	Outpatients and healthy controls	99 (51)	7–18
Uran et al.^c^ [64]	2015	Turkey	Conference abstract	Clinical	Prospective	patients and healthy controls	99 (51)	7–18
Althoff et al. [9]	2016	USA	Regular article	Population-based	Prospective	Population	6483 (51)	13–18
Averna et al. [65]	2016	Italy	Case report	Case study	Retrospective	Outpatient	1 (0)	11
Baweja et al. [66]	2016	USA	Regular article	Clinical	Prospective^2^	Outpatients	38 (28)	7–12
Brotman et al. [67]	2016	USA	Conference abstract	Clinical	Prospective	Patients and healthy controls	110 (45)	9–19
Carlson et al. [68]	2016	USA	Conference abstract	Clinical	Prospective	Community	36 (56)	6, 9 and 12
Copeland et al. [37]	2016	USA	Conference abstract	Clinical	Prospective^2^	Community	112 (NA)	M 11.4
Dougherty et al.^d^ [69]	2016	USA	Regular article	Population-based	Prospective	Population	473 (46)	3 6 and 9
Freeman et al. [70]	2016	USA	Regular article	Clinical	Prospective^2^	Outpatients	597 (39)	6–18
Fristad et al. [71]	2016	USA	Regular article	Clinical	Prospective	Patients	217 (38)	6–12
Gold et al. [72]	2016	USA	Regular article	Clinical	Prospective	Community, outpatients and healthy controls	184 (40)	8–19
Kessel et al. [39]	2016	USA	Regular article	Population-based	Prospective	Community	373 (45)	9
Kilic et al. [73]	2016	Turkey	Case report	Case study	Retrospective	Outpatient	1 (0)	18
Mitchell et al.^a^ [74]	2016	Canada	Regular article	Clinical	Prospective	Outpatients	108 (68)	13–19
Mulraney et al.^b^ [75]	2016	Australia	Regular article	Clinical	Prospective	Community	179 (25)	6–8
Pogge et al. [76]	2016	USA	Conference abstract	Clinical	Prospective	Inpatients	100 (NA)	6–12
Stoddard et al. [77]	2016	USA	Regular article	Clinical	Prospective	Patients and healthy controls	89 (48)^3^	8–18
Stoddard^e^ [78]	2016	USA	Conference abstract	Clinical	Prospective	Patients and healthy Controls	115 (44)	8–17
Taskiran et al. [79]	2016	Turkey	Conference abstract	Clinical	Prospective	Outpatients	29 (NA)	M 9.2
Tiwari et al. [80]	2016	India	Regular article	Clinical	Prospective	Inpatients	70 (24)	6–16
Topal et al.^f^ [81]	2016	Turkey	Conference abstract	Clinical	Prospective	Outpatients	90 (48)	12–16
Topal et al.^f^ [82]	2016	Turkey	Conference abstract	Clinical	Prospective	Offspring of parents with mood disorder	87 (43)	12–16
Tudor et al. [83]	2016	USA	Case report	Case study	Retrospective	Patients	1 (100)	9
Tufan et al. [84]	2016	Turkey	Regular article	Clinical	Retrospective	Outpatients	403 (NA)	6–17
Wiggins et al. [41]	2016	USA	Regular article	Clinical	Prospective	Outpatients and healthy controls	71 (40)	9–21
Alexander et al. [85]	2017	USA	Conference abstract	Population-based	Prospective	Population	500 (NA)	5–21
Dougherty et al.^d^ [86]	2017	USA	Regular article	Clinical	Prospective	Community	329 (51)	6 and 9
Estrada Prat et al. [87]	2017	Spain	Regular article	Clinical	Prospective	Patients	35 (33)	6–18
Eyre et al.^g^ [88]	2017	UK	Regular article	Clinical	Prospective	Patients	696 (16)	6–18
Faheem et al. [89]	2017	USA	Regular article	Clinical	Retrospective	Inpatients	490 (NA)	6–18
Higdon et al. [90]	2017	USA	Conference abstract	Clinical	Prospective	Overweight patients	438 (52)	7–19
Jain [91]	2017	India	Conference abstract	Clinical	Prospective	Patients	25 (12)	6–9
Jalnapurkar et al. [92]	2017	USA	Conference abstract	Clinical	Prospective	Inpatients	95 (NA)	8–17
Kircanski et al.^h^ [93]	2017	USA	Conference abstract	Clinical	Prospective	Outpatients	197 (46)	8–18
Kircanski et al.^h^ [94]	2017	USA	Conference abstract	Clinical	Prospective	Outpatients and healthy controls	199 (54)	8–18
Le et al. [95]	2017	USA	Conference abstract	Clinical	Retrospective	Patients	7268 (NA)	< 18
Martin et al. [96]	2017	USA	Regular article	Clinical	Prospective	Outpatients	139 (25)	4–5
Matthews et al. [97]	2017	USA	Conference abstract	Clinical	Prospective	Previous inpatients	91 (43)	6–17
McTate et al. [53]	2017	USA	Case report	Case study	Prospective	Outpatient	1 (100)	9
Mitchell et al. [98]	2017	Australia	Conference abstract	Clinical	Prospective	Youth at familial risk of BD and controls	242 (NA)	12–30
Munhoz et al.^i^ [99]	2017	Brazil	Regular article	Cohort study	Prospective	Birth cohort (Pelotas study)	3490 (48)	11
Özyurt et al. [100]	2017	Turkey	Regular article	Clinical	Retrospective	Outpatients	12 (0)	8–17
Pagliaccio et al. [101]	2017	USA	Regular article	Clinical	Prospective	Patients and healthy controls	83 (48)	8–18
Perepletchikova et al. [102]	2017	USA	Regular article	Clinical	Prospective	Community and outpatients	43 (44)	7–12
Perhamus et al. [103]	2017	USA	Conference abstract	Clinical	Prospective	Patients and healthy controls	120 (45)	8–18
Propper et al. [104]	2017	Canada	Regular article	Clinical	Prospective	Offspring of parents with BD or MDD	180 (53)	6–18
Ramires et al. [105]	2017	Brazil	Case report	Case study	Retrospective	Outpatients	1 (0)	7
Stoddard et al.^e^ [106]	2017	USA	Regular article	Clinical	Prospective	patients	115 (44)	8–17
Stoddard et al. [107]	2017	USA	Conference abstract	Clinical	Prospective	Patients and healthy controls	42 (42)	8–21
Swetlitz et al. [108]	2017	USA	Conference abstract	Clinical	Prospective	Outpatients and healthy controls	48 (58)	8–17
Taskiran et al.^j^ [109]	2017	Turkey	Conference abstract	Clinical	Prospective	Patients and healthy controls	43 (NA)	M 9.5
Taskiran et al.^j^ [110]	2017	Turkey	Conference abstract	Clinical	Prospective	Patients and healthy controls	43 (NA)	NA
Tseng et al.^k^ [111]	2017	USA	Conference abstract	Clinical	Prospective	Patients and healthy controls	197 (59)	8–18
Waxmonsky et al. [112]	2017	USA	Conference abstract	Clinical	Retrospective	Outpatients	56 (29)	7–12
Abouzed et al. [113]	2018	Egypt	Conference abstract	Clinical	Prospective	Offspring of parents with ADHD and healthy controls	212 (NA)	6–18
Bryant et al. [114]	2018	USA	Conference abstract	Clinical	Retrospective	Patients	360 (29)	4–17
Cuffe et al. [115]	2018	USA	Conference abstract	Population-based	Prospective	Student population	292 (48)	5–17
de la Peña et al. [38]	2018	Latin America^1^	Regular article	Clinical	Prospective	Outpatients	80 (40)	6–18
Delaplace et al. [116]	2018	France	Regular article	Clinical	Prospective	Outpatients	21 (10)	9–15
Fridson et al. [117]	2018	USA	Conference abstract	Clinical	Retrospective	Patients	839 (NA)	6–18
Grau et al. [36]	2018	Germany	Regular article	Population-based	Prospective	Population	2413 (NA)	18–94
Kircanski et al.^h^ [118]	2018	USA	Regular article	Clinical	Prospective	Community	197 (46)	8–18
Miller et al. [119]	2018	USA	Regular article	Clinical	Prospective	outpatients	19 (42)	12–17
Mroczkowski et al. [120]	2018	USA	Regular article	Juvenile justice	Retrospective	Juvenile justice involved youths	2266 (30)	8–18
Pan et al. [121]	2018	Taiwan	Regular article	Clinical	Prospective	Outpatients	58 (17)	7–17
Sagar-Ouriaghli et al. [122]	2018	Great Britain	Regular article	Clinical	Prospective^2^	Outpatients	117 (NA)	6–12
Vidal-Ribas et al. [123]	2018	USA	Regular article	Clinical	Prospective	Outpatients and healthy controls	116 (38)	8–20
Walyzada et al. [124]	2018	USA	Conference abstract	Clinical	Retrospective	Outpatients	1088 (46)	NA
Wiggins et al. [125]	2018	USA	Regular article	Clinical	Prospective	Outpatients	425 (51)	3–5
Winters et al. [126]	2018	USA	Regular article	Clinical	Prospective	Patients	22 (31)	9–15
Basu et al. [127]	2019	Australia	Regular article	Clinical	Retrospective	Patients	101 (58)	6–12
Benarous et al. [128]	2019	France	Case report	Case study	Retrospective	Inpatients	6 (30)	10–14
Benarous et al. [129]	2019	France	Conference abstract	Clinical	Retrospective	Outpatients	163 (40)	7–17
Chen et al. [130]	2019	Taiwan	Regular article	Population-based	Prospective	Population	4816 (48)	10–17
Eyre et al.^g^ [131]	2019	UK	Regular article	Clinical	Prospective	Patients	696 (16)	6–18
Guilé [132]	2019	France	Conference abstract	Clinical	Prospective	Patients and healthy controls	21 (100)	M 11.7 ± 3 SD
Haller et al. [133]	2019	USA	Conference abstract	Clinical	Prospective	Patients and healthy controls	44 (43)	8–17
Ignaszewski et al. [134]	2019	USA	Case report	Case study	Retrospective	Outpatient	1 (0)	14
Linke et al. [135]	2019	USA	Case report	Case study	Retrospective	Outpatient	1 (0)	11
Linke et al. [136]	2019	USA	Regular article	Clinical	Prospective	Patients and healthy controls	118 (46)	11–21
Mulraney et al. [137]	2019	Australia	Conference abstract	Cohort study	Prospective	Patients	134 (28)	7–10
Rice et al. [138]	2019	USA	Case report	Case study	Retrospective	Inpatient	1 (100)	12
Towbin et al. [139]	2019	USA	Regular article	Clinical	Prospective	Patients	53 (36)	7–17
Tseng et al.^k^ [140]	2019	USA	Regular article	Clinical	Prospective	Patients and healthy controls	195 (50)	8–18
Tüğen et al. [141]	2019	Turkey	Regular article	Population-based	Prospective	Community	356 (55)	6–11
Ünal et al. [40]	2010	Turkey	Regular article	Clinical	Prospective	Outpatients	120 (49)	6–17
Alexander et al. [27]	2020	USA	Regular article	Clinical	Prospective	Community	523 (41)	6–17
Benarous et al. [142]	2020	France	Regular article	Clinical	Prospective	Patients	30 (29)	6–16
Benarous et al. [143]	2020	France, Canada	Regular article	Clinical	Retrospective	outpatients	163 (43)	7–27
Chang et al. [144]	2020	Taiwan	Regular article	Clinical	Prospective	Patients	101 (31)	7–18
Cimino et al. [145]	2020	Italy	Regular article	Clinical	Prospective	Patients and healthy controls	150 (48)	8–9
Haller et al. [146]	2020	USA	Conference abstract	Clinical	Prospective	Patients	189 (34)	M 13.1
Haller et al. [147]	2020	USA	Regular article	Clinical	Prospective	Patients	98 (41)	7–17
Johnstone et al. [148]	2020	USA	Regular article	Clinical	Retrospective	Patients	168 (23)	6–12
Laporte et al.^i^ [45]	2020	Brazil	Regular article	Cohort study	Prospective	Birth cohort (Pelotas study)	3562 (NA)	10–12
Le et al. [149]	2020	USA	Regular article	Population-based	Retrospective	Patients covered by Medicaid	814,919 (49)	< 18
Tseng et al. [150]	2020	USA	Conference abstract	Clinical	Prospective	Patients	69 (NA)	M 14.5

*DMDD* disruptive mood dysregulation disorder, *ADHD* attention deficit hyperactivity disorder, *ODD* oppositional defiant disorder, *BD* bipolar disorder, *SMD* severe mood dysregulation, *MDD* major depressive disorder. *NA* information not available

^a^Mitchell et al. (2015) and (2016) report data from overlapping samples

^b^Schipzand et al. (2015) and Mulraney et al. (2016) report data from overlapping samples

^c^Uran et al. (2015) abstract and regular article report on same data

^d^ Dougherty et al. (2016) and (2017) partly report on overlapping data

^e^Stoddard et al. (2016) and (2017) report on overlapping data

^f^ Topal et al. (2016) abstracts report data from overlapping samples

^g^Eyre et al. (2017) and (2019) report on overlapping data

^h^Kircanski et al. (2017) and (2018) report on overlapping data

^i^Munhoz et al. (2017) and Laporte et al. (2020) report on overlapping data

^j^Taskiran et al. (2017) abstracts report on overlapping data

^k^Tseng et al. (2017) and (2019) report on overlapping data

^1^Mexico, Colombia, Chile, and Uruguay

^2^DMDD diagnosis was obtained retrospectively

^3^Where not otherwise specified, patients were in- and outpatients

^4^Experiment 1

^5^Mean (M) is given, where no information about range was available

There was an initial increase in numbers of publications from 2013 until 2017, after which numbers dropped again: *k* = 1 study in 2013 (0.9%; *k* = 0 abstracts), *k* = 5 in 2014 (4.5%; *k* = 0 abstracts), *k* = 8 in 2015 (7.3%; *k* = 6 abstracts, 5.5%), *k* = 24 in 2016 (21.8%; *k* = 7 abstracts, 6.4%), *k* = 29 in 2017 (26.4%; *k* = 16 abstracts, 14.5%), *k* = 16 in 2018 (14.5%; *k* = 5 abstracts, 4.5%), and *k* = 16 in 2019 (14.5%; *k* = 4 abstracts, 3.6%) and *k* = 11 in 2020 (10.0%; *k* = 2 abstracts, 1.8%).

Most of the included studies stem from the United States of America (*k* = 66, 60.0%; *k* = 26 abstracts, 23.6%). Other countries of origin include Turkey (*k* = 12, 10.9%; *k* = 6 abstracts, 5.5%), France (*k* = 6, 5.5%; *k* = 2 abstracts, 1.8%), Australia (*k* = 5, 4.5%; *k* = 3 abstracts, 2.7%), Brazil (*k* = 3, 2.7%; *k* = 0 abstracts), Canada (*k* = 3, 2.7%; *k* = 1 abstracts), United Kingdom (*k* = 3, 2.7%; *k* = 0 abstracts), Taiwan (*k* = 3, 2.7%; *k* = 0 abstracts), India (*k* = 2, 1.8%; *k* = 1 abstract), Spain (*k* = 2, 1.8%; *k* = 1 abstract), Italy (*k* = 2, 1.8%; *k* = 0 abstracts), Egypt (*k* = 1, 0.9%; *k* = 1 abstract) and Germany (*k* = 1, 0.9%; *k* = 0 abstracts). *K* = 1 regular article includes data from Mexico, Colombia, Chile, and Uruguay (0.9%).

Most study samples consisted of patients (in- and/or outpatients) *k* = 85 (77.3%; *k* = 33 abstracts, 30.0%). Of those, some reported to include only outpatients (*n* = 39, 35.5%; *k* = 10 abstracts, 9.1%), or inpatients (*n* = 7, 6.4%; *k* = 3 abstracts, 2.7%). Further, study samples consisted of community (*n* = 10, 9.1%; *k* = 2 abstracts, 1.8%), population (*n* = 7, 6.4%; *k* = 2 abstracts, 1.8%), youth at familial risk of psychiatric disorders (*n* = 4, 3.6%; *k* = 3 abstracts, 2.7%) birth cohorts (*n* = 2, 1.8%; *k* = 0 abstracts), juvenile justice involved youths (*n* = 1, 0.9%; *k* = 0 abstracts), and overweight patients (*n* = 1, 0.9%, *k* = 1 abstract). Many of the studies further examined healthy controls in addition to a patient group (*n* = 26, 23.6%). Sample sizes ranged from *k* = 1 in case-reports to *k* = 6483 in a large population-based study. Examined ages lay between 2 and 94 years of age, while most samples’ ages ranged from early school-age to adolescence or young adulthood.

### Measurement of DMDD diagnosis

A variety of instruments were used to diagnose DMDD in the included studies. The instrument used most often was the *Kiddie Schedule for Affective Disorders and Schizophrenia Present and Lifetime Version*, K-SADS-PL [19] (*n* = 48, 43.6%; *k* = 20 abstracts, 18.2%) in combination with the DMDD module (Table 2), *k* = 27 (24.5%; *k* = 12 abstracts, 10.9%). The *Preschool Age Psychiatric Assessment*, PAPA [20] was used in *k* = 7 studies (6.4%; *k* = 1 abstracts, 0.9%), of which *k* = 4 did so in combination with ODD and depression sections. In *k* = 3 (2.7%) studies each, the *Child and Adolescent Psychiatric Assessment*, CAPA [21] (*n* = 0 abstracts), the *Diagnostic Interview Schedule for Children, Version IV*, DISC-IV [22] (*n* = 1 abstract, 0.9%), and the *Washington University in St. Louis Kiddie Schedule for Affective Disorders and Schizophrenia,* WASH-U-K-SADS [23] (*n* = 1 abstract, 0.9%) were used. In *k* = 2 studies (1.8%) each, the Breton, Bergeron and Labelle DMDD Scale [24] (*n* = 1 abstract, 0.9%), the Conners rating scales [25] (*n* = 1 abstract, 0.9%), the *Development and Well-Being Assessment*, DAWBA [26] and the *Extended Strengths and Weaknesses Assessment of Normal Behavior*, E‐SWAN [27] (*n* = 1 abstracts, 0.9%) were used. Instruments used in *k* = 1 (0.9%) regular articles each included the *Child and Adolescent Symptom Inventory,* CASI [28], the *Child Behavior Check List dysregulation profile*, CBCL-DP [29], the *Children’s Interview for Psychiatric Syndromes*, ChIPS [30] in combination with the *Mini-International Neuropsychiatric Interview* for Children and Adolescents, MINI-KID [31], the *Composite International Diagnostic Interview* CIDI [32], the *Diagnostic Infant and Preschool Assessment*, DIPA [33], the Mandarin Version of the K-SADS-Epidemiological Version for DSM-5, K-SADS-E [34], the *Structured Clinical Interview for DSM-IV*, SCID-IV [35], a self-created set of six questions [36], and the *Voice Diagnostic Interview Schedule for Children,* V-DISC [22]. A not otherwise specified structured interview was reported in *k* = 1 conference abstract [37].

**Table 2:** Measurement of DMDD in studies included in the systematic review, by tool

Authors	Year	Main diagnostic DMDD measure	Additional measures or specifications	Rater	Were psychometric properties for DMDD measure assessed in this study?^a^
Benarous et al. [128]	2019	K-SADS-PL	DMDD module	Clinician	No
Benarous et al. [142]	2020	K-SADS-PL	DMDD module	Clinician	No
Brotman et al. [67]	2016	K-SADS-PL	DMDD module	Clinician	Raters trained to IRR, κ ≥ 0.9; ICCs ≥ .9 differentiating DMDD module from mania/hypomania
Gold et al. [72]	2016	K-SADS-PL	DMDD module	Clinician	Raters trained to IRR, κ ≥ 0.9; ICCs ≥ .9 differentiating DMDD module from mania/hypomania
Haller et al. [133]	2019	K-SADS-PL	DMDD module	Clinician	Raters trained to IRR, κ ≥ 0.9; ICCs ≥ .9 differentiating DMDD module from mania/hypomania
Haller et al	2020	K-SADS-PL	DMDD module	Clinician	No
Kircanski et al. [93, 94]	2017	K-SADS-PL	DMDD module	Clinician	Raters trained to IRR, κ ≥ 0.9; ICCs ≥ .9 differentiating DMDD module from mania/hypomania
Kircanski et al. [118]	2018	K-SADS-PL	DMDD module	Clinician	Raters trained to IRR, κ ≥ 0.9; ICCs ≥ .9 differentiating DMDD module from mania/hypomania
Kircanski et al. [93, 94]	2017	K-SADS-PL	DMDD module	Clinician	Raters trained to IRR, κ ≥ 0.9; ICCs ≥ .9 differentiating DMDD module from mania/hypomania
Linke et al. [135]	2019	K-SADS-PL	DMDD module	Clinician	Raters trained to IRR, κ ≥ 0.9; ICCs ≥ .9 differentiating DMDD module from mania/hypomania
Linke et al. [136]	2019	K-SADS-PL	DMDD module	Clinician	Raters trained to IRR, κ ≥ 0.9; ICCs ≥ .9 differentiating DMDD module from mania/hypomania
Pagliaccio et al. [101]	2017	K-SADS-PL	DMDD module	Clinician	Raters trained to IRR, κ ≥ 0.9; ICCs ≥ .9 differentiating DMDD module from mania/hypomania
Perepletchikova et al. [102]	2017	K-SADS-PL	DMDD module	Clinician	No
Propper et al. [104]	2017	K-SADS-PL	DMDD module	Clinician	No
Stoddard et al. [77]	2016	K-SADS-PL	DMDD module	Clinician	Raters trained to IRR, κ ≥ 0.9; ICCs ≥ .9 differentiating DMDD module from mania/hypomania
Swetlitz et al. [108]	2017	K-SADS-PL	DMDD module	Clinician	Raters trained to IRR, κ ≥ 0.9; ICCs ≥ .9 differentiating DMDD module from mania/hypomania
Tseng et al. [62]	2015	K-SADS-PL	DMDD module	Clinician	Raters trained to IRR, κ ≥ 0.9; ICCs ≥ .9 differentiating DMDD module from mania/hypomania
Tseng et al. [111]	2017	K-SADS-PL	DMDD module	Clinician	Raters trained to IRR, κ ≥ 0.9; ICCs ≥ .9 differentiating DMDD module from mania/hypomania
Tseng et al. [140]	2019	K-SADS-PL	DMDD module	Clinician	Raters trained to IRR, κ ≥ 0.9; ICCs ≥ .9 differentiating DMDD module from mania/hypomania
Tudor et al. [83]	2016	K-SADS-PL	DMDD module	Clinician	NA
Vidal-Ribas et al. [123]	2018	K-SADS-PL	DMDD module	Clinician	Raters trained to IRR, κ ≥ 0.9; ICCs ≥ .9 differentiating DMDD module from mania/hypomania
Perhamus et al. [103]	2017	K-SADS-PL	DMDD module	Clinician	Raters trained to IRR, κ ≥ 0.9; ICCs ≥ .9 differentiating DMDD module from mania/hypomania
Stoddard [78]	2016	K-SADS-PL	DMDD module	Clinician	Raters trained to IRR, κ ≥ 0.9; ICCs ≥ .9 differentiating DMDD module from mania/hypomania
Stoddard et al. [61]	2015	K-SADS-PL	DMDD module	Clinician	Raters trained to IRR, κ ≥ 0.9; ICCs ≥ .9 differentiating DMDD module from mania/hypomania
Stoddard et al. [106]	2017	K-SADS-PL	DMDD module	Clinician	Raters trained to IRR, κ ≥ 0.9; ICCs ≥ .9 differentiating DMDD module from mania/hypomania
Stoddard et al. [107]	2017	K-SADS-PL	DMDD module	Clinician	Raters trained to IRR, κ ≥ 0.9; ICCs ≥ .9 differentiating DMDD module from mania/hypomania
Tüğen et al. [141]	2019	K-SADS-PL	Considering changes based on DSM-5; CBCL as a pre-screening	Clinician	No
Sagar-Ouriaghli et al. [122]	2018	K-SADS-PL	Elaborate system of filters to check all DSM-5 criteria	Clinician	NA
Freeman et al. [70]	2016	K-SADS-PL	Mood modules from WASH-U-KSADS; retrospective rating based on DSM-5 criteria	Clinician	No
Estrada Prat et al. [87]	2017	K-SADS-PL	ODD module	Clinician	No
Mitchell et al. [59]	2015	K-SADS-PL	ODD module and narrative summaries	Clinician	No
Mitchell et al. [74]	2016	K-SADS-PL	ODD module as well as narrative summaries (for DMDD criteria A-G)	Clinician	No
Winters et al. [126]	2018	K-SADS-PL	Querying parent and child about DMDD criteria posted on the DSM-5 website	Clinician	No
Deveney et al. [57]	2015	K-SADS-PL	Retrospectively applied DMDD criteria to prospectively obtained K-SADS-PL SMD module	Clinician	Raters trained to IRR, κ ≥ 0.9; ICCs ≥ .9 differentiating DMDD module from mania/hypomania
Topal et al. [81]	2016	K-SADS-PL	Screening of DSM-5 criteria	Clinician	Inter-rater agreement for DMDD symptoms and diagnosis was high, Tau = 0.76, p = 0.00
Topal et al. [82]	2016	K-SADS-PL	SMD module and screening for DSM-5 criteria	Clinician	Inter-rater agreement for DMDD symptoms and diagnosis was high, Tau = 0.76, p = 0.00
Estrada-Prat et al. [58]	2015	K-SADS-PL	SMD module	Clinician	No
Miller et al. [119]	2018	K-SADS-PL	SMD module	Clinician	No
Mitchell et al. [98]	2017	K-SADS-PL	SMD module	Clinician	NA
Özyurt et al. [100]	2017	K-SADS-PL	SMD module	Clinician	No
Towbin et al. [139]	2019	K-SADS-PL	SMD module	Clinician	Raters trained to IRR, κ ≥ 0.9; ICCs ≥ .9 differentiating DMDD module from mania/hypomania
Uran et al. [63]	2015	K-SADS-PL	SMD module	Clinician	NA
de la Peña et al. [38]	2018	K-SADS-PL	Spanish version modified under the DSM-5 criteria	Clinician	Cronbach's alpha for DMDD = 0.92
Roy et al. [43]	2014	K-SADS-PL	Teacher rating scales	Clinician	NA
Abouzed et al. [113]	2018	K-SADS-PL		Clinician	NA
Higdon et al. [90]	2017	K-SADS-PL		Clinician	No
Uran et al. [64]	2015	K-SADS-PL		Clinician	NA
Ünal et al. [40]	2019	K-SADS-PL-DSM-5-T	Turkish adaptation of K-SADS-PL including DMDD module	Clinician	IRR *κ* = 0.63; consensus validity 96% consensus, *κ* = 0.70; concurrent validity with ARI
Wiggins et al. [41]	2016	K-SADS	In youths under age 18; SCID-III-R in youths over age 18, with the DMDD module	Clinician	Raters trained to IRR, κ ≥ 0.9; ICCs ≥ .9 differentiating DMDD module from mania/hypomania
Taskiran et al. [109, 110]	2017	K-SADS		Clinician	NA
Cimino et al. [145]	2020	Clinical diagnosis	Based on DSM-5 criteria	Clinician	No
Le et al. [95]	2017	Clinical diagnosis	Based on DSM-5 criteria	Clinician	No
Pan et al. [121]	2018	Clinical diagnosis	Based on DSM-5 criteria	Clinician	No
Tiwari et al. [80]	2016	Clinical diagnosis	Based on DSM-5 criteria	Clinician	No
Ignaszewski et al. [134]	2019	Clinical diagnosis	Based on parent and child report and behavior seen longitudinally across course of treatment	Clinician	No
Ramires et al. [105]	2017	Clinical diagnosis	Parent and child interviews, CBCL, Rorschach method, teacher report form	Clinician	No
Bryant et al. [114]	2018	Clinical diagnosis	Retrospective based on medical records	Clinician	No
Rice et al. [138]	2019	Clinical diagnosis		Clinician	No
Benarous et al. [129]	2019	Chart review	Checklist for symptoms of temper dysregulation disorder with dysphoria	Clinician	No
Benarous et al. [143]	2020	Chart review	Checklist for symptoms of temper dysregulation disorder with dysphoria	Clinician	No
Pogge et al. [76]	2016	Chart review	Checklist of the variables corresponding to DSM-5 criteria	Clinician	NA
Fridson et al. [117]	2018	Chart review	electronic medical record review	Clinician	NA
Basu et al. [127]	2019	Chart review	Self-created symptom check list	Clinician	No
Faheem et al. [89]	2017	Chart review		Clinician	No
Walyzada et al. [124]	2018	Chart review		Clinician	No
Dougherty et al. [86]	2017	PAPA	K-SADS-PL after age 6	Clinician	IRR for all diagnoses and symptom scales *κ* = 0.64–0.89; ICC = 0.71–0.97
Carlson et al. [68]	2016	PAPA	K-SADS-PL at age 9 and 12	Clinician	NA
Wiggins et al. [125]	2018	PAPA	K-SADS-PL in a subset of reassessed children	Clinician	*κ* = 0.83 to 1.00 on all interviews
Copeland et al. [10]	2013	PAPA	ODD and depression sections	Clinician	NA
Dougherty et al. [54]	2014	PAPA	ODD and depression sections	Clinician	IRR for all diagnoses and symptom scales *κ* = 0.64–0.89; ICC = 0.71–0.97
Dougherty et al. [69]	2016	PAPA	ODD and depression sections	Clinician	IRR for all diagnoses and symptom scales *κ* = 0.64–0.89; ICC = 0.71–0.97
Kessel et al. [39]	2016	PAPA	ODD and depression sections	Clinician	ICC for dimensional lifetime psychopathology symptom scores ranged from 0.86 to 0.97. Cronbach's alpha = 0.75
Copeland et al. [11]	2014	CAPA	Conduct problems and depression sections	Clinician	NA
Eyre et al. [88]	2017	CAPA	ODD and depression sections	Clinician	Raters trained to IRR, κ ≥ 0.9; ICCs ≥ .9 differentiating DMDD module from mania/hypomania
Eyre et al. [131]	2019	CAPA	ODD and depression sections	Clinician	Raters trained to IRR, κ ≥ 0.9; ICCs ≥ .9 differentiating DMDD module from mania/hypomania
Mulraney et al. [75]	2016	DISC-IV	ODD and MDD modules	Clinician	No
Schilpzand et al. [60]	2015	DISC-IV	ODD and MDD modules	Clinician	No
Cuffe et al. [115]	2018	DISC-IV	Three study stages: 1. Screening 2. DISC-IV and 3. K-SADS-PL	Clinician	No
Fristad et al. [71]	2016	WASH-U-KSADS	ODD supplement	Clinician	No
Waxmonsky et al. [112]	2017	WASH-U-KSADS	Disruptive Behavior Disorders Structured Parent Interview	Clinician	IRR κ > 0.9
Baweja et al. [66]	2016	WASH-U-KSADS	Disruptive Behavior Disorders Structured Parent Interview	Clinician	No
Guilé [132]	2019	Breton, Bergeron & Labelle DMDD Scale	Self- and informant-based questionnaire	Self-rating	No
Delaplace et al. [116]	2018	Breton, Bergeron & Labelle DMDD Scale	Self- and informant-based questionnaire, and K-SADS-PL with DMDD module	Self-rating/clinician	NA
Tufan et al. [84]	2016	Conners	8th (ready to pick up a fight, quick to anger) and 21st (is cranky and sullen) items and further details from screening instruments; subset of patients' caregivers interviewed about DMDD symptoms via phone	Clinician	κ = 0.68
Mulraney et al. [137]	2019	Conners	DISC-IV	Clinician	No
Laporte et al. [45]	2020	DAWBA	DMDD section	Clinician	NA
Munhoz et al. [99]	2017	DAWBA	DMDD section	Clinician	No
Alexander et al. [27]	2020	E-SWAN	DMDD module	Parent-rating	Cronbach's alpha = 0.98, AUC 0.85
Alexander et al. [85]	2017	E-SWAN		Parent-rating	Reliabilities range from .77 to .96
Le et al. [149]	2020	Case records	Medicaid records	Clinician	No
Averna et al. [65]	2016	CBCL-DP	Anxious/Depressed, Attention Problems, and Aggressive Behaviour syndrome scales	Clinician	NA
McTate et al. [53]	2017	ChIPS and MINI-KID	Both measures were checked for relevant items	Clinician	NA
Johnstone et al. [148]	2020	CASI	DMDD subscale	Parent-rating	No
Althoff et al. [9]	2016	CIDI	Strengths and Difficulties section of the PSAQ	Clinician	Rates of this new measure were compared with other psychiatric diagnoses and to service usage, no numbers reported
Martin et al. [96]	2017	DIPA	ODD and MDD modules	Clinician	NA
Chen et al. [130]	2019	K-SADS-E	In Mandarin	Clinician	Reported in Chen et al. 2017
Sparks et al. [56]	2014	SCID-IV	Sections from K-SADS-PL and ODD module, and review of narrative summaries of clinical presentations	Clinician	NA
Grau et al. [36]	2018	Set of questions	Six questions referring to current severe temper outbursts and severe temper outburst during primary school to determine whether DSM-5 criteria were met	Self-rating	NA
Copeland et al. [37]	2016	Structured interview	Structured diagnostic interview completed with a parent; diagnosis of DMDD was made post hoc because its criteria overlapped entirely with those of ODD and depression	Clinician	NA
Mroczkowski et al. [120]	2018	V-DISC	ODD module	Self-rating/clinician	No

*K* = 10 studies without any information about DMDD measurement or psychometric properties are not shown

*DMDD* disruptive mood dysregulation disorder, *ADHD* attention deficit hyperactivity disorder, *ODD* oppositional defiant disorder, *BD* bipolar disorder, *SMD* severe mood dysregulation, *MDD* major depressive disorder, *ARI* Affective Reactivity Index, *NA* information not available

^a^If not provided in the publication, this information was obtained through direct contact with study authors

In *k* = 8 studies (7.3%; *k* = 2 abstracts, 1.8%) a clinical diagnosis was made without any specific measures and in *k* = 7 studies (6.4%; *k* = 3 abstracts, 2.7%) diagnosis was made using chart review or Medicaid records (*n* = 1). Finally, *k* = 10 (9.1%; *k* = 6 abstracts, 5.5%) studies did not provide any information on the measure used.

In most of the measures used in the included studies, a clinician rated the patients’ and participants’ statements and behavior (*n* = 91, 82.7%), while others consisted of a parent- (*n* = 3, 2.7%), or self-rating (*n* = 4, 3.6%). No information about the rater was given in *k* = 10 (9.1%) studies.

### Psychometric properties

In *k* = 79 studies (71.8%; *k* = 17 abstracts, 15.5%), any information on the presence or absence of psychometric properties of the measure used to diagnose DMDD was given or obtained from the authors. Of those, in *k* = 39 (35.5%; *k* = 4 abstracts, 3.6%) no psychometric properties have been obtained or reported as part of the study or using the study data. In the remaining *k* = 40 studies (36.4%, *k* = 13 abstracts, 11.8%), the most commonly reported psychometric property was reliability, with *k* = 33 (30.0%; *k* = 13 abstracts, 11.8%) reporting inter-rater reliability ranging from *κ* = 0.6 to 1 and *k* = 29 (26.4%; *k* = 11 abstracts, 10.0%) reporting intra-class correlation coefficients. Three studies assessed internal consistency with Cronbach's alpha = 0.92 for a Spanish version of the K-SADS-PL modified under the DSM-5 to diagnose DMDD [38], and Cronbach's alpha = 0.75 for the PAPA [39] and 0.98 for the E-SWAN DMDD scale [27]. In the studies of the NIMH group around Dr. Ellen Leibenluft (*n* = 25, 22.7%), raters were trained to reach inter-rater reliability with *κ* ≥ 0.9, before they contributed to interviews/data collection for the respective studies. Cases were further discussed in conference with other reliable clinicians and in a lab meeting where leading clinicians reviewed the core criteria before diagnosis was made. The same group also provided ICCs ≥ 0.9 differentiating the DMDD module from the mania/hypomania part of the K-SADS-PL. One study examined consensus validity between a clinical psychiatric interview based on DSM-5 diagnostic criteria and the Turkish version of the DSM-5 version of the K-SADS-PL (K-SADS-PL-DSM-5-T), led by two independent clinician-researchers [40]. A consensus of 96%, *κ* = 0.63 was reached. Further, concurrent validity was evaluated with the Affective Reactivity Index (ARI), *κ* = 0.70. One study generated Receiver Operating Characteristic (ROC) curves to obtain Area Under the Curve (AUC) for their diagnostic instrument, as a measure of predictive validity. With an AUC value of 0.85, the E-SWAN DMDD scale performed equally well in predicting diagnoses compared to the Affective Reactivity Index [27].

## Discussion

Evidence from this systematic review points to a variety of different measures used for the evaluation and diagnosis of DMDD. The majority of studies used clinician-rated structured interviews in combination with DMDD specific symptom checklists. Few studies employed questionnaires or interviews specifically designed to measure DMDD or its severity. In the following, some of the most used measures are presented in more detail, before practical aspects, such as available languages and cost as well as diagnostic challenges and future directions are discussed.

**Fig. 2:**
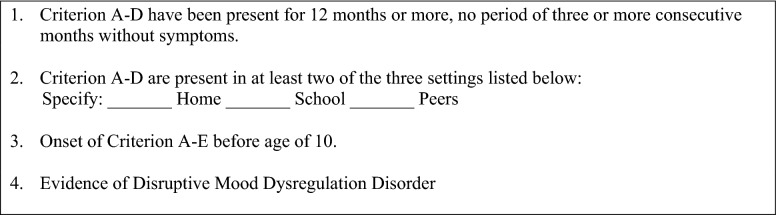
K-SADS-PL DMDD module. Each of the questions are evaluated with 0, 1 or 2 for current and/or past episodes. The diagnostic criteria of DMDD are listed below the questions in the module (see supplementary material for the DSM-5 diagnostic criteria). Reprint authorized by Joan Kaufman, owner of the copyright of the K-SADS-PL

By far the most often used instrument was the K-SADS-PL in combination with the DMDD module. The K-SADS-PL is a semi-structured interview to diagnose mental disorders in children aged 6–18. Administration time is estimated to be about 75 min for psychiatric patients and 35–45 min for healthy control subjects. It is freely available for download online. It has high inter-rater reliability and good to excellent test–retest reliability [19]. The DMDD module has been developed by a workgroup around Leibenluft, in collaboration with the K-SADS developer Kaufman. A prior version of this module was based on a research diagnosis coined severe mood dysregulation (SMD) [4]. The DMDD module is a checklist consisting of four items probing for the DSM-5 criteria to be met (Fig. 2, see supplementary material for the DSM-5 diagnostic criteria A-K). With training and case discussion, the module can be administered with high inter-rater reliability [41]. It has further shown to differentiate well between other mood disorders such as mania/hypomania.

Our study’s findings revealed different methodological approaches to diagnosing DMDD. Some of the instruments utilized in the reviewed studies consisted of a symptom checklist. This was the case not only for the K-SADS-PL DMDD module but also for its precursor, the SMD module or the ODD module. While the checklist format might suggest simplicity, it is most often used in the context of the more comprehensive K-SADS-PL semi-structured interview, which is used by raters to create a proxy diagnostic using a combination of ODD, depression, or mania criteria, and thereby empirically derive a DMDD diagnosis. Moreover, a combination of comprehensive structured or semi-structured interviews (e.g., K-SADS-PL, SCID, DISC or CIDI) and self-made checklists or clinical evaluation to probe for DSM criteria have been employed. An approach that has further been adopted in some of the reviewed studies was to search established interviews or questionnaires (CBCL-DP, Conners, ChIPS, MINI or PAPA/CAPA/DIPA) for items relevant to the DMDD diagnosis. This approach likely stems from the fact that these studies assessed DMDD retrospectively in data not collected with the focus of determining the prevalence of DMDD.

Few instruments have been deliberately designed to diagnose DMDD. Those identified by this systematic review were the K-SADS-PL DMDD module, the Breton, Bergeron and Labelle DMDD scale (available as a semi-structured interview and questionnaire), the E-SWAN DMDD module (interview) and the DAWBA DMDD section (interview; see Table 3 for an overview of instruments designed to diagnose DMDD). The instruments contain 4–34 items assessing occurrences, frequencies, and circumstances of temper tantrums/outbursts and irritable or angry mood. All instruments are available in the English language. The Breton, Bergeron and Labelle DMDD Scale is additionally available in French, and the DAWBA DMDD section additionally exists in Danish and Portuguese. The E-SWAN and DAWBA scales are freely available online or upon request to the authors. Indicated age ranges are similar, encompassing preschool age to early adulthood. While the K-SADS-PL DMDD module, the Breton, Bergeron and Labelle DMDD Scale, and the DAWBA DMDD section provide categorical outcomes, the E-SWAN DMDD module is designed to capture DMDD symptoms dimensionally. This scale reconceptualizes each diagnostic criterion for DMDD as a behavior, which can range from high (strengths) to low (weaknesses). Regarding the psychometric properties, it seems that the DMDD module has been evaluated most often, as high levels of reliability are reported in many studies. However, these reliabilities have been reached artificially by training raters to differentiate K-SADS-PL DMDD from mania modules. Although useful for the clinic, this approach does not correspond to the evaluation of reliability as a measure of consistency between raters for a certain diagnostic instrument used in a study. Therefore, a more comprehensive psychometric evaluation of this widely used measure is necessary. Besides the DMDD module, psychometric properties have been reported for the E-SWAN DMDD module. The reliability of this scale has been reported to be excellent (Cronbach’s alpha = 0.98). Reporting of psychometric properties of the other DMDD scales is still pending. Studies using tools to diagnose DMDD followed a broad spectrum of study objectives and hypotheses. Thus, the DMDD measure and its psychometric properties might not have been the focus of attention, which might be the reason for not providing this information. However, to determine gold-standard measurement, psychometric evaluation of the currently used diagnostic measures is necessary.

**Table 3:** Instruments designed to diagnose DMDD

	Method	Number of items	Freely available/costs	Languages available	Outcome dimensional	Indicated age range
K-SADS-PL DMDD module	Symptom Checklist	4	Yes	English	No	6–19
Breton, Bergeron & Labelle DMDD Scale	Semi-structured interview/questionnaire	11	NA	English, French	No	NA
DAWBA DMDD section	Interview	34	Yes	English, Danish, Portugese	No	5–18
E-SWAN DMDD module	Interview	10	Yes	English	Yes	6–17

*DMDD* disruptive mood dysregulation disorder, *NA* information not available

When assessing the psychometric properties of the instruments used in the included studies, mainly measures of reliability have been considered and reported. However, the psychometric evaluation of a diagnostic tool ideally also contains the assessment of its validity. Neither content-related (e.g., construct validity, factorial structure) nor criterion-related types of validity (e.g., concurrent or predictive validity) have been considered broadly in existing studies. One study reported substantial consensus validity (*κ* = 0.63) and concurrent validity (*κ* = 0.70) of a Turkish version of the K-SADS-PL [40]. A further study showed substantial predictive validity of the E-SWAN DMDD module (AUC = 0.85) [27]. Consequently, measures of validity require more attention in future research on the measurement of DMDD and should guide the reporting of respective measures in future studies.

Given the aim of the present systematic review, to provide an overview of existing instruments for the assessment of DMDD and their use in the diagnostic process, we refrained from conducting a formal risk of bias assessment of included studies. The potential risk of bias does not interfere with the aim of the present review and was thus deemed irrelevant.

Since the advent of DMDD, clinicians and researchers have noted various challenges and the diagnosis is not without controversy [17]. The characteristic symptoms of DMDD, namely irritable mood and temper outbursts are observed across multiple disruptive behavior and mood disorders and the validity of DMDD as a distinct diagnosis has been questioned [13, 42, 43]. Further, DMDD could not be distinguished from ODD based on symptomatology alone in a population-based study [44]. It has further been criticized that alternative thresholds for defining DMDD, as well as a closer investigation of clinically relevant thresholds, have so far only partly been considered in the existing literature [45]. The lack of precision in diagnosing DMDD might in part account for the criticism voiced about the clinical entity of DMDD. Similarly, the heterogeneity in measurement of DMDD up to date, as found in the present systematic review of the literature, might account for variations in current prevalence and comorbidity rates as well as findings on associations with risk factors or functional outcomes in individuals with DMDD. Studies designed a priori with appropriate instruments to capture DMDD are therefore necessary [46].

While the diagnostic entity of DMDD may be a useful clinical heuristic, many researcher-clinicians focus their efforts on broader transdiagnostic constructs, such as irritability [8]. Irritability has been defined as a heightened proneness to anger relative to peers [47, 48] which can be seen as a personality trait with a continuous distribution across the population. In children and adolescents with DMDD, by definition, irritability is severe and expressed stably across time. In the last decade, there has been a marked increase in irritability research and there have been neuroscientific as well as treatment-related approaches to understanding pathophysiological mechanisms [41, 49]. Until now, whether persistent irritability between temper outbursts and the outbursts themselves are independent of each other, or whether the mood between outbursts is rather a concatenation of less severe tantrums, remains unknown.

In addition to further psychometric evaluation of current diagnostic measures and the development of a gold-standard diagnostic measure, adjuvant measurement approaches have become popular in the last decade. One promising approach to describe the full spectrum of irritability and temper outbursts in patients’ everyday lives is ecological momentary assessment (EMA; also known as experience sampling method or ambulatory assessment). This involves the repeated sampling of patients’ experiences or mood, performed via a handheld device such as a mobile phone. This measurement method has high ecological validity, avoiding biases due to retrospective assessments [50]. The repeated measurement of affect, with multiple measurements during the day over several days, potentially in children or their parents might be insightful in the characterization of hourly and daily fluctuations of mood in patients with irritability and/or DMDD.

To inform the debate around the diagnostic entity of DMDD, the application of Research Domain Criteria (RDoC) constructs may yield greater clarity in terms of underlying processes and thus inform nosology as well as appropriate interventions [51]. The constructs of frustrative non-reward (Negative Valence Domain), reward prediction error (Positive Valence domain), attention and language (Cognitive domain) as well as arousal (Arousal and Regulatory systems) have been found to be particularly promising in this regard.

## Limitations of the review

The present systematic review encompasses literature involving instruments for the categorical diagnosis of DMDD. In view of the described developments regarding dimensional aspects of DMDD, a systematic review of the literature on dimensional constructs, such as irritability would be informative and topical. Similarly, a comprehensive overview on the examination of developmentally non-appropriate temper tantrums would be of interest in this regard.

A substantial proportion of the studies included in this systematic review stems from one laboratory in the United States. More studies evaluating the reliability and validity of the DMDD diagnosis should be conducted in other laboratories, to reduce the potential bias of findings and address cultural differences.

Psychological assessment should not be made based on any one instrument in isolation. Rather, test findings should be integrated with information from personal and educational histories and in collaboration with other clinicians [52, 53]. Consequently, using any current instruments to evaluate DMDD will require additional query and clinical evaluation. For research purposes, however, standardized assessment methods are inevitable.

## Conclusion and future directions

A variety of different measures have been used for the evaluation of DMDD. The most commonly used and established instrument consists of a symptom checklist, while more recently developed structured interviews and questionnaires are still to establish their reliability and validity in diagnosing DMDD. Dimensional and experimental approaches to assessing irritability and temper outbursts as well as their interrelation might bring forth more clarity about DMDD symptomatology in children.

## Supplementary Information

Below is the link to the electronic supplementary material.

Supplementary file1 (DOCX 13 kb)

Supplementary file2 (DOC 64 kb)

## Data Availability

Can be requested from the corresponding author.

## References

[1] American Psychiatric Association (2013). Diagnostic and statistical manual of mental disorders.

[2] Leibenluft E (2011). Severe mood dysregulation, irritability, and the diagnostic boundaries of bipolar disorder in youths. Am J Psychiatry.

[3] Stringaris A, Vidal-Ribas P, Brotman MA, Leibenluft E (2018). Practitioner review: definition, recognition, and treatment challenges of irritability in young people. J Child Psychol Psychiatry.

[4] Leibenluft E, Charney DS, Towbin KE (2003). Defining clinical phenotypes of juvenile mania. Am J Psychiatry.

[5] Adleman NE, Kayser R, Dickstein D (2011). Neural correlates of reversal learning in severe mood dysregulation and pediatric bipolar disorder. J Am Acad Child Adolesc Psychiatry.

[6] Deveney CM, Connolly ME, Jenkins SE (2012). Neural recruitment during failed motor inhibition differentiates youths with bipolar disorder and severe mood dysregulation. Biol Psychol.

[7] Thomas LA, Brotman MA, Muhrer EJ (2012). Parametric modulation of neural activity by emotion in youth with bipolar disorder, youth with severe mood dysregulation, and healthy volunteers. Arch Gen Psychiatry.

[8] Brotman MA, Kircanski K, Stringaris A (2017). Irritability in youths: a translational model. Am J Psychiatry.

[9] Althoff RR, Crehan ET, He J-P (2016). Disruptive mood dysregulation disorder at ages 13–18: results from the National Comorbidity Survey—Adolescent Supplement. J Child Adolesc Psychopharmacol.

[10] Copeland WE, Angold A, Costello EJ, Egger H (2013). Prevalence, comorbidity, and correlates of DSM-5 proposed disruptive mood dysregulation disorder. Am J Psychiatry.

[11] Copeland WE, Shanahan L, Egger H (2014). Adult diagnostic and functional outcomes of DSM-5 disruptive mood dysregulation disorder. Am J Psychiatry.

[12] Tapia V, John RM (2018). Disruptive mood dysregulation disorder. J Nurse Pract.

[13] Axelson D, Findling RL, Fristad MA (2012). Examining the proposed disruptive mood dysregulation disorder diagnosis in children in the longitudinal assessment of manic symptoms study. J Clin Psychiatry.

[14] Mayes SD, Waxmonsky J, Calhoun SL (2015). Disruptive mood dysregulation disorder (DMDD) symptoms in children with autism, ADHD, and neurotypical development and impact of co-occurring ODD, depression, and anxiety. Res Autism Spectr Disord.

[15] Mayes SD, Waxmonsky JD, Calhoun SL, Bixler EO (2016). Disruptive mood dysregulation disorder symptoms and association with oppositional defiant and other disorders in a general population child sample. J Child Adolesc Psychopharmacol.

[16] De Rosa C (2018) ICD-11 sessions in the 17th World Congress of Psychiatry. World Psychiatry. 17(1):119–120. 10.1002/wps.20507 10.1002/wps.20507 PMC5775136 29352549

[17] Bruno A, Celebre L, Torre G (2019). Focus on disruptive mood dysregulation disorder: a review of the literature. Psychiatry Res.

[18] Moher D, Liberati A, Tetzlaff J (2009). Preferred reporting items for systematic reviews and meta-analyses: the PRISMA statement. PLoS Med.

[19] Kaufman J, Birmaher B, Brent D (1997). Schedule for affective disorders and schizophrenia for school-age children-present and lifetime version (K-SADS-PL): initial reliability and validity data. J Am Acad Child Adolesc Psychiatry.

[20] Egger H, Angold A, DelCarmen-Wiggins R, Carter A (2004). The preschool age psychiatric assessment (PAPA): a structured parent interview for diagnosing psychiatric disorders in preschool children. Handbook of Infant and Toddler Mental Health Assessment.

[21] Angold A, Costello EJ (2000). The Child and Adolescent Psychiatric Assessment (CAPA). J Am Acad Child Adolesc Psychiatry.

[22] Shaffer D, Fisher P, Lucas CP (2000). NIMH Diagnostic Interview Schedule for Children Version IV (NIMH DISC-IV): description, differences from previous versions, and reliability of some common diagnoses. J Am Acad Child Adolesc Psychiatry.

[23] Geller B, Zimerman B, Williams M (2001). Reliability of the Washington University in St. Louis Kiddie Schedule for Affective Disorders and Schizophrenia (WASH-U-KSADS) Mania and Rapid Cycling Sections. J Am Acad Child Adolesc Psychiatry.

[24] Boudjerida A, Labelle R, Bergeron L et al (2018) Disruptive mood dysregulation disorder scale in adolescence. 23rd World Congress of the International Association for Child & Adolescent Psychiatry and Allied Professions, Prague

[25] Conners CK (2008) Conners 3rd edition manual. In: Multi-health systems, Toronto Ontario, Canada

[26] Goodman R, Ford T, Richards H (2000). The development and well-being assessment: description and initial validation of an integrated assessement of child and adolescent psychopathology. J Child Psychol Psychiatry.

[27] Alexander LM, Salum GA, Swanson JM, Milham MP (2020). Measuring strengths and weaknesses in dimensional psychiatry. J Child Psychol Psychiatr.

[28] Gadow KD, Sprafkin JN (2015) Child and Adolescent Symptoms Inventory, vol 5. In: Checkmate Plus, Stony Brook New York.

[29] Althoff RR, Rettew DC, Ayer LA, Hudziak JJ (2010). Cross-informant agreement of the Dysregulation Profile of the Child Behavior Checklist. Psychiatry Res.

[30] Weller EB, Weller RA, Fristad MA (2000). Children’s interview for psychiatric syndromes (ChIPS). J Am Acad Child Adolesc Psychiatry.

[31] Sheehan DV, Lecrubier Y, Sheehan KH (1998). The Mini-International Neuropsychiatric Interview (M.I.N.I): The development and validation of a structured diagnostic psychiatric interview for DSM-IV and ICD-10. J Clin Psychiatry.

[32] Kessler RC, Ustün TB (2004). The World Mental Health (WMH) Survey Initiative Version of the World Health Organization (WHO) Composite International Diagnostic Interview (CIDI). Int J Methods Psychiatr Res.

[33] Scheeringa MS, Haslett N (2010). The reliability and criterion validity of the Diagnostic Infant and Preschool Assessment: a new diagnostic instrument for young children. Child Psychiatry Hum Dev.

[34] Chen Y-L, Shen L-J, Gau SS-F (2017). The Mandarin version of the Kiddie-Schedule for Affective Disorders and Schizophrenia-Epidemiological version for DSM-5—a psychometric study. J Formos Med Assoc.

[35] First MB, Spitzer RL, Williams JBW (1995). Structured clinical interview for DSM-IV (SCID).

[36] Grau K, Plener PL, Hohmann S (2018). Prevalence rate and course of symptoms of disruptive mood dysregulation disorder (DMDD): a population-based study. Z Kinder Jugendpsychiatr Psychother.

[37] Copeland WE, Simonoff E, Stringaris A (2016). Disruptive mood dysregulation disorder in children with autism spectrum disorder. J Am Acad Child Adolesc Psychiatry.

[38] de la Peña FR, Rosetti MF, Rodríguez-Delgado A (2018). Construct validity and parent–child agreement of the six new or modified disorders included in the Spanish version of the Kiddie Schedule for Affective Disorders and Schizophrenia present and Lifetime Version DSM-5 (K-SADS-PL-5). J Psychiatr Res.

[39] Kessel EM, Dougherty LR, Kujawa A (2016). Longitudinal associations between preschool disruptive mood dysregulation disorder symptoms and neural reactivity to monetary reward during preadolescence. J Child Adolesc Psychopharmacol.

[40] Ünal F, Öktem F, Çetin Çuhadaroglu F (2019). Reliability and validity of the schedule for affective disorders and schizophrenia for school-age children-present and lifetime version, DSM-5 November 2016-Turkish Adaptation (K-SADS-PL-DSM-5-T). Turk J Psychiatry.

[41] Wiggins JL, Brotman MA, Adleman NE (2016). Neural correlates of irritability in disruptive mood dysregulation and bipolar disorders. Am J Psychiatry.

[42] Lochman JE, Evans SC, Burke JD (2015). An empirically based alternative to DSM-5’s disruptive mood dysregulation disorder for ICD-11. World Psychiatry.

[43] Roy AK, Lopes V, Klein RG (2014). Disruptive mood dysregulation disorder (DMDD): a new diagnostic approach to chronic irritability in youth. Am J Psychiatry.

[44] Mayes SD, Waxmonsky JD, Calhoun SL, Bixler EO (2016) Disruptive Mood Dysregulation Disorder Symptoms and Association with Oppositional Defiant and Other Disorders in a General Population Child Sample. J Child Adolesc Psychopharmacol 26(2):101–106. 10.1089/cap.2015.0074 10.1089/cap.2015.0074 PMC4800381 26745442

[45] Laporte PP, Matijasevich A, Munhoz TN (2020). Disruptive mood dysregulation disorder: symptomatic and syndromic thresholds and diagnostic operationalization. J Am Acad Child Adolesc Psychiatry.

[46] Tseng W-L (2020). Editorial: A transdiagnostic symptom requires a transdiagnostic approach: neural mechanisms of pediatric irritability. J Am Acad Child Adolesc Psychiatry.

[47] Leibenluft E, Stoddard J (2013). The developmental psychopathology of irritability. Dev Psychopathol.

[48] Vidal-Ribas P, Brotman MA, Valdivieso I (2016). The status of irritability in psychiatry: a conceptual and quantitative review. J Am Acad Child Adolesc Psychiatry.

[49] Haller SP, Stoddard J, MacGillivray C (2018). A double-blind, randomized, placebo-controlled trial of a computer-based Interpretation Bias Training for youth with severe irritability: a study protocol. Trials.

[50] Larson R, Csikszentmihalyi M (1992) Validity and reliability of the Experience Sampling Method. In: Csikszentmihalyi M, Vries M (eds) The Experience of Psychopathology: Investigating Mental Disorders in their Natural Settings (pp. 43- 57). Cambridge: Cambridge University Press. 10.1017/CBO9780511663246.006

[51] Meyers E, DeSerisy M, Roy AK (2017). Disruptive mood dysregulation disorder (DMDD): an RDoC perspective. J Affect Disord.

[52] Matarazzo JD (1990). Psychological assessment versus psychological testing. Validation from Binet to the school, clinic, and courtroom. Am Psychol.

[53] McTate EA, Leffler JM (2017). Diagnosing disruptive mood dysregulation disorder: integrating semi-structured and unstructured interviews. Clin Child Psychol Psychiatry.

[54] Dougherty LR, Smith VC, Bufferd SJ (2014). DSM-5 disruptive mood dysregulation disorder: correlates and predictors in young children. Psychol Med.

[55] Parmar A, Vats D, Parmar R, Aligeti M (2014). Role of naltrexone in management of behavioral outbursts in an adolescent male diagnosed with disruptive mood dysregulation disorder. J Child Adolesc Psychopharmacol.

[56] Sparks GM, Axelson DA, Yu H (2014). Disruptive mood dysregulation disorder and chronic irritability in youth at familial risk for bipolar disorder. J Am Acad Child Adolesc Psychiatry.

[57] Deveney CM, Hommer RE, Reeves E (2015). A prospective study of severe irritability in youths: 2- and 4-year follow-up. Depress Anxiety.

[58] Estrada Prat X, Alvarez Guerrico I, Camprodon Rosanas E (2015). Disruptive mood dysregulation disorder and pediatric bipolar disorder. Sleep and attention. Eur Child Adolesc Psych.

[59] Mitchell RHB, Hlastala SA, Mufson L, et al (2015) Correlates of disruptive mood dysregulated disorder (DMDD) phenotype among adolescents with bipolar disorder. 17th Annual Conference of the International Society for Bipolar Disorders, June 3​–6, Toronto, Canada. Bipolar Disorders, Volume 17, S1. 10.1111/bdi.12309

[60] Schilpzand EJ, Hazell P, Nicholson J, et al (2015) Comorbidity and correlates of disruptive mood dysregulation disorder in 6–8 year old children with ADHD. 5th World Congress on ADHD: From Child to Adult Disorder: 28th-31st May, Glasgow Scotland. Atten Defic Hyperact Disord. Volume 7, Suppl 1:1-119. 10.1007/s12402-015-0169-y

[61] Stoddard J, Sharif-Askary B, Harkins E (2015). Preliminary evidence for computer-based training targeting hostile interpretation bias as a treatment for DMDD. Neuropsychopharmacology.

[62] Tseng W-L, Brotman M, Deveney C (2015). Neural mechanisms of irritability in youth across diagnoses: dimensional and categorical approaches. Neuropsychopharmacology.

[63] Uran P, Kılıç BG (2015). Family Functioning, Comorbidities, and behavioral profiles of Children with ADHD and disruptive mood dysregulation disorder. J Atten Disord.

[64] Uran P, Kilic B (2015). Comparison of family functioning and psychiatric comorbidities of children with attention deficit hyperactivity disorder and disruptive mood dysregulation disorder. Eur Child Adolesc Psychiatry.

[65] Averna R, D’Agati E, Vicari S (2016). Low-dose aripiprazole monotherapy in a young child with disruptive mood dysregulation disorder. Ther Adv.

[66] Baweja R, Belin PJ, Humphrey HH (2016). The effectiveness and tolerability of central nervous system stimulants in school-age children with attention-deficit/hyperactivity disorder and disruptive mood dysregulation disorder across home and school. J Child Adolesc Psychopharmacol.

[67] Brotman MA, Tseng W-L, Wiggins J et al (2016) Neural correlates of attention bias in irritability and anxiety. The American College of Neuropsychopharmacology (ACNP) 56th Annual Meeting Poster Session, December 3–7 2017, Volume 42. 10.1038/npp.2017.264

[68] Carlson GA, Barrios CS, Dougherty LR, Klein DN (2016). Stability and predictors of disruptive mood dysregulation disorder in young children. J Am Acad Child Adolesc Psychiatry.

[69] Dougherty LR, Smith VC, Bufferd SJ (2016). Disruptive mood dysregulation disorder at the age of 6 years and clinical and functional outcomes 3 years later. Psychol Med.

[70] Freeman AJ, Youngstrom EA, Youngstrom JK, Findling RL (2016). Disruptive mood dysregulation disorder in a community mental health clinic: prevalence, comorbidity and correlates. J Child Adolesc Psychopharmacol.

[71] Fristad MA, Wolfson H, Algorta GP (2016). Disruptive mood dysregulation disorder and bipolar disorder not otherwise specified: fraternal or identical twins?. J Child Adolesc Psychopharmacol.

[72] Gold AL, Brotman MA, Adleman NE (2016). Comparing brain morphometry across multiple childhood psychiatric disorders. J Am Acad Child Adolesc Psychiatry.

[73] Kilic O, Demirbas Cakir E, Tufan AE (2016). Disruptive mood dysregulation disorder in adults: a case report. Eur psychiatr.

[74] Mitchell RHB, Timmins V, Collins J (2016). Prevalence and correlates of disruptive mood dysregulation disorder among adolescents with bipolar disorder. J Child Adolesc Psychopharmacol.

[75] Mulraney M, Schilpzand EJ, Hazell P (2016). Comorbidity and correlates of disruptive mood dysregulation disorder in 6–8-year-old children with ADHD. Eur Child Adolesc Psychiatry.

[76] Pogge DL, Chase D, Buccolo M et al (2016) Prevalence of and comorbidities with disruptive mood dysregulation disorder in an inpatient setting. The Scientific Proceedings of the 63th Annual Meeting of the American Academy of Child & Adolescent Psychiatry Journal of the American Academy of Child & Adolescent Psychiatry, Volume 55, Issue 10S

[77] Stoddard J, Sharif-Askary B, Harkins EA (2016). An open pilot study of training hostile interpretation bias to treat disruptive mood dysregulation disorder. J Child Adolesc Psychopharmacol.

[78] Stoddard J (2016). Transdiagnostic neural mechanisms of irritability. J Am Acad Child Adolesc Psychiatry.

[79] Taskiran S, Mutluer T, Sanli I (2016). The correlation between distractorincorporated continuous performance test and neuropsychological test battery results of children with attentiondeficit/hyperactivity disorder. J Am Acad Child Adolesc Psychiatry.

[80] Tiwari R, Agarwal V, Arya A (2016). An exploratory clinical study of disruptive mood dysregulation disorder in children and adolescents from India. Asian J Psychiatr.

[81] Topal Z, Demir N, Tuman TC (2016). Rates of disruptive mood dysregulation disorder among adolescent offspring of parents with recurrent major depressive disorder versus those with bipolar disorder and matched healthy controls. J Am Acad Child Adolesc Psychiatry.

[82] Topal Z, Demir N, Tuman TC (2016). Rates of disruptive mood dysregulation disorder in adolescent children of parents with recurrent depression or bipolar disorder and healthy controls. Klinik Psikofarmakoloji Bulteni.

[83] Tudor ME, Ibrahim K, Bertschinger E (2016). Cognitive-behavioral therapy for a 9-year-old girl with disruptive mood dysregulation disorder. Clin Case Stud.

[84] Tufan E, Topal Z, Demir N (2016). Sociodemographic and clinical features of disruptive mood dysregulation disorder: a chart review. J Child Adolesc Psychopharmacol.

[85] Alexander LM, Salum GA, Swanson JM, Milham MP (2017) Development of the Extended Strengths and Weaknesses Assessment of Normal Behavior Rating Scale (E-SWAN). The Scientific Proceedings of the 64th Annual Meeting of the American Academy of Child & Adolescent Psychiatry Journal of the American Academy of Child & Adolescent Psychiatry, Volume 56, Issue 10S

[86] Dougherty LR, Barrios CS, Carlson GA, Klein DN (2017). Predictors of later psychopathology in young children with disruptive mood dysregulation disorder. J Child Adolesc Psychopharmacol.

[87] Estrada Prat X, Álvarez-Guerrico I, Bleda-Hernández MJ (2017). Sleep study in disruptive mood dysregulation disorder and bipolar children. Actas Esp Psiquiatr.

[88] Eyre O, Langley K, Stringaris A (2017). Irritability in ADHD: associations with depression liability. J Affect Disord.

[89] Faheem S, Petti V, Mellos G (2017). Disruptive mood dysregulation disorder and its effect on bipolar disorder. Ann Clin Psychiatry.

[90] Higdon C, Fornari V, Sheridan E, et al (2017) Conducting a multi-site, community-based, pragmatic research trial: study design, recruitment barriers, and initial sample characteristics of mobility. The Scientific Proceedings of the 64th Annual Meeting of the American Academy of Child & Adolescent Psychiatry Journal of the American Academy of Child & Adolescent Psychiatry, Volume 56, Issue 10S

[91] Jain U (2017) The use of guanfacine (Intuniv XR) in the treatment of disruptive mood dysregulation disorder—Clinical experience from telepsychiatry. 25th European Congress of Psychiatry, European Psychiatry, Volume 41S, S1-S910. 10.1016/j.eurpsy.2017.01.449

[92] Jalnapurkar IR, Desai P, Pemberton AM (2017). Stressors and aggressors: violent aggression often precedes inpatient admission and exacerbates caregiver stress. J Am Acad Child Adolesc Psychiatry.

[93] Kircanski K, White L, Tseng W-L, et al (2017) Computational phenotyping reveals a double dissociation in the neural mechanisms of irritability and anxiety in youth. The American College of Neuropsychopharmacology (ACNP) 56th Annual Meeting Poster Session, December 3–7 2017, Volume 42. 10.1038/npp.2017.264

[94] Kircanski K, White L, Tseng W-L, et al (2017) Shared and unique neural correlates of threat processing in pediatric irritability and anxiety. 72nd Annual Scientific Convention and Meeting of Biological Psychiatry, Journal of Biological Psychiatry Volume 81, Issue 10S

[95] Le JF, Lohr WD, Feygin YB et al (2017) Examining trends and interactions in the diagnoses of pediatric bipolar disorders and disruptive mood dysregulation disorder (DMDD) in Kentucky Children and Adolescents Receiving Medicaid. The Scientific Proceedings of the 64th Annual Meeting of the American Academy of Child & Adolescent Psychiatry Journal of the American Academy of Child & Adolescent Psychiatry, Volume 56, Issue 10S

[96] Martin SE, Hunt JI, Mernick LR (2017). Temper loss and persistent irritability in preschoolers: implications for diagnosing disruptive mood dysregulation disorder in early childhood. Child Psychiatry Hum Dev.

[97] Matthews DT, Matthews GW (2017) Disruptive mood dysregulation disorder: Medical management without the use of antipsychotics. 2016 NEI Psychopharmacology Congress. CNS Spectrums 22(1):62–109. 10.1017/S1092852916000900

[98] Mitchell PB, Perich T, Frankland A (2017). Irritability and disruptive mood dysregulation disorder as potential precursors of bipolar disorder. Bipolar Disorders.

[99] Munhoz TN, Santos IS, Barros AJD (2017). Perinatal and postnatal risk factors for disruptive mood dysregulation disorder at age 11: 2004 Pelotas Birth Cohort Study. J Affect Disord.

[100] Özyurt G, Emiroglu N, Baykara B, Akay Pekcanlar A (2017). Effectiveness and adverse effects of methylphenidate treatment in children diagnosed with disruptive mood dysregulation disorder and attention-deficit hyperactivity disorder: a preliminary report. Psychiatry Clin Psychopharmacol.

[101] Pagliaccio D, Wiggins JL, Adleman NE (2017). Behavioral and neural sustained attention deficits in disruptive mood dysregulation disorder and attention-deficit/hyperactivity disorder. J Am Acad Child Adolesc Psychiatry.

[102] Perepletchikova F, Nathanson D, Axelrod SR (2017). Randomized clinical trial of dialectical behavior therapy for preadolescent children with disruptive mood dysregulation disorder: feasibility and outcomes. J Am Acad Child Adolesc Psychiatry.

[103] Perhamus G, Kircanski K, Lee Wiggins J et al (2017) Face emotion labeling in pediatric irritability: behavioral and neural correlates. The Scientific Proceedings of the 64th Annual Meeting of the American Academy of Child & Adolescent Psychiatry Journal of the American Academy of Child & Adolescent Psychiatry, Volume 56, Issue 10S

[104] Propper L, Cumby J, Patterson VC (2017). Disruptive mood dysregulation disorder in offspring of parents with depression and bipolar disorder. Br J Psychiatry.

[105] Ramires VRR, Godinho LBR, Goodman G (2017). The therapeutic process of a child diagnosed with disruptive mood dysregulation disorder. Psychoanal Psychol.

[106] Stoddard J, Tseng W-L, Kim P (2017). Association of irritability and anxiety with the neural mechanisms of implicit face emotion processing in youths with psychopathology. JAMA Psychiat.

[107] Stoddard J, Jones M, Haller S (2017). Identifying the mechanisms of interpretation bias in irritability. J Am Acad Child Adolesc Psychiatry.

[108] Swetlitz C, Averbeck B, Leibenluft E (2017). Explore-exploit decision making: differences in information-seeking behavior in pediatric psychopathology. J Am Acad Child Adolesc Psychiatry.

[109] Taskiran S, Mutluer T, Necef I (2017). Neuropsychological profile differences between children with disruptive mood dysregulation disorder (DMDD) and attention-deficit/hyperactivity disorder (ADHD): a preliminary study. J Am Acad Child Adolesc Psychiatry.

[110] Taskiran S, Turkakin E, Karamanci C (2017). Face emotion recognition differences with respect to frustration in disruptive mood dysregulation disorder (DMDD) and attention-deficit/hyperactivity disorder (ADHD). J Am Acad Child Adolesc Psychiatry.

[111] Tseng W-L, Deveney C, Brotman M et al (2017) Neural mechanisms of frustration and irritability across diagnoses. 72nd Annual Scientific Convention and Meeting of Biological Psychiatry, Journal of Biological Psychiatry Volume 81, Issue 10S.

[112] Waxmonsky JG, Waschbusch D, Babocsai L, Belin P (2017). Assessment and treatment of hostile attribution bias in children with disruptive mood dysregulation disorder. J Am Acad Child Adolesc Psychiatry.

[113] Abouzed M, Elawady A (2018) Disruptive mood dysregulation disorder in offspring of parents with ADHD. E-Poster Walk. European Psychiatry, 48(S1):S141-S358. 10.1016/j.eurpsy.2017.12.016

[114] Bryant B, Bear MH, Rowlett JK (2018) DMDD patients with and without a history of childhood abuse and/or neglect: comparison of hospital length of stay, use of antipsychotics, and restraints. The Scientific Proceedings of the 65th Annual Meeting of the American Academy of Child & Adolescent Psychiatry, Journal of the American Academy of Child & Adolescent Psychiatry, Volume 57, Issue 10S

[115] Cuffe SP, Glassman RS, Singh K, Holbrook J (2018) Identification of bipolar DMDD in a school-based study of school-aged children and adolescents. The Scientific Proceedings of the 65th Annual Meeting of the American Academy of Child & Adolescent Psychiatry, Journal of the American Academy of Child & Adolescent Psychiatry, Volume 57, Issue 10S

[116] Delaplace R, Garny de La Rivière S, Bon Saint Come M (2018). Sleep and disruptive mood dysregulation disorder: a pilot actigraphy study. Arch Pediatr.

[117] Fridson R, Bailey S, Edwards E, et al (2018) Comparison of prescribed pharmacotherapy of patients diagnosed with DMDD versus bipolar disorder in child and adolescent psychiatric outpatients. The Scientific Proceedings of the 65th Annual Meeting of the American Academy of Child & Adolescent Psychiatry, Journal of the American Academy of Child & Adolescent Psychiatry, Volume 57, Issue 10S

[118] Kircanski K, White LK, Tseng W-L (2018). A latent variable approach to differentiating neural mechanisms of irritability and anxiety in youth. JAMA Psychiat.

[119] Miller L, Hlastala SA, Mufson L (2018). Interpersonal psychotherapy for mood and behavior dysregulation: pilot randomized trial. Depress Anxiety.

[120] Mroczkowski MM, McReynolds LS, Fisher P, Wasserman GA (2018) Disruptive mood dysregulation disorder in juvenile justice. J Am Acad Psychiatry Law 46:329–338. 10.29158/JAAPL.003767-18 10.29158/JAAPL.003767-18 30368465

[121] Pan P-Y, Fu A-T, Yeh C-B (2018). Aripiprazole/methylphenidate combination in children and adolescents with disruptive mood dysregulation disorder and attention-deficit/hyperactivity disorder: an open-label study. J Child Adolesc Psychopharmacol.

[122] Sagar-Ouriaghli I, Milavic G, Barton R (2018). Comparing the DSM-5 construct of Disruptive Mood Dysregulation Disorder and ICD-10 Mixed disorder of emotion and conduct in the UK Longitudinal Assessment of manic symptoms (UK-LAMS) Study. Eur Child Adolesc Psychiatry.

[123] Vidal-Ribas P, Brotman MA, Salum GA (2018). Deficits in emotion recognition are associated with depressive symptoms in youth with disruptive mood dysregulation disorder. Depress Anxiety.

[124] Walyzada F, Manocha P, Odom C et al (2018) Prescribing practices of antipsychotics in children and adolescents with a diagnosis of DMDD in an outpatient setting. Journal of the American Academy of Child & Adolescent Psychiatry, 579(10):Supplement p. S170. 10.1016/j.jaac.2018.09.123.

[125] Wiggins JL, Briggs-Gowan MJ, Estabrook R (2018). Identifying clinically significant irritability in early childhood. J Am Acad Child Adolesc Psychiatry.

[126] Winters DE, Fukui S, Leibenluft E, Hulvershorn LA (2018). Improvements in irritability with open-label methylphenidate treatment in youth with comorbid attention deficit/hyperactivity disorder and disruptive mood dysregulation disorder. J Child Adolesc Psychopharmacol.

[127] Basu S, Isaacs AN (2019). Profile of transcultural patients in a regional Child and Adolescent Mental Health Service in Gippsland, Australia: the need for a multidimensional understanding of the complexities. Int J Soc Psychiatry.

[128] Benarous X, Ferrafiat V, Zammit J (2019). Effective use of atomoxetine to treat six inpatient youths with disruptive mood dysregulation disorder without attention deficit disorder. CNS Spectr.

[129] Benarous X (2019) Are youths with DMDD different from youths with other depressive disorders? A retrospective chart review. The Scientific Proceedings of the 66th Annual Meeting of the American Academy of Child & Adolescent Psychiatry Journal of the American Academy of Child & Adolescent Psychiatry, Volume 58, Issue 10

[130] Chen Y-L, Chen WJ, Lin K-C (2019). Prevalence of DSM-5 mental disorders in a nationally representative sample of children in Taiwan: methodology and main findings. Epidemiol Psychiatr Sci.

[131] Eyre O, Riglin L, Leibenluft E (2019). Irritability in ADHD: association with later depression symptoms. Eur Child Adolesc Psychiatry.

[132] Guilé J-M (2019). Sleep abnormalities in disruptive mood dysregulation disorder: an actigraphy study. J Am Acad Child Adolesc Psychiatry.

[133] Haller SP, Jones M, Pine D, Leibenluft E, Brotman MA, Stoddard J (2019) A computational model to measure mechanisms of interpretation bias training for treating disruptive mood dysregulation disorder. Biological Psychiatry 85(10)Suppl:S69. 10.1016/j.biopsych.2019.03.180

[134] Ignaszewski MJ, Munshi K, Fogler J, Augustyn M (2019). Transitions, suicidality, and underappreciated autism spectrum disorder in a high school student. J Dev Behav Pediatrics.

[135] Linke JO, Kircanski K, Brooks J (2019). Exposure-based cognitive-behavioral therapy for disruptive mood dysregulation disorder: an evidence-based case study. Behav Ther.

[136] Linke JO, Adleman NE, Sarlls J (2019). White matter microstructure in pediatric bipolar disorder and disruptive mood dysregulation disorder. J Am Acad Child Adolesc Psychiatry.

[137] Mulraney M, Silk TJ, Efron D et al (2019) Persistence and neural correlates of disruptive mood dysregulation disorder in 10-year-old children with ADHD. 7th World Congress on ADHD: From Child to Adult Disorder: 25th-28th April, Lisbon Portugal. Atten Defic Hyperact Disord; Volume 11, Suppl 1:1-89. 10.1007/s12402-019-00295-7 10.1007/s12402-019-00295-7 30941637

[138] Rice T, Simon H, Barcak D (2019). Amantadine for treatment of disruptive mood dysregulation disorder symptoms. J Child Adolesc Psychopharmacol.

[139] Towbin K, Vidal-Ribas P, Brotman MA (2019). A double-blind randomized placebo-controlled trial of citalopram adjunctive to stimulant medication in youth with chronic severe irritability. J Am Acad Child Adolesc Psychiatry.

[140] Tseng W-L, Deveney CM, Stoddard J (2019). Brain mechanisms of attention orienting following frustration: associations with irritability and age in youths. Am J Psychiatry.

[141] Tüğen LE, Göksu M, Burcu Ayaz A (2020). Disruptive mood dysregulation disorder in a primary school sample. Asian J Psychiatr.

[142] Benarous X, Bury V, Lahaye H (2020). Sensory processing difficulties in youths with disruptive mood dysregulation disorder. Front Psychiatry.

[143] Benarous X, Renaud J, Breton JJ (2020). Are youths with disruptive mood dysregulation disorder different from youths with major depressive disorder or persistent depressive disorder?. J Affect Disord.

[144] Chang C-H, Chang Y-C, Cheng H, Tzang R-F (2020). Treatment efficacy of internet gaming disorder with attention deficit hyperactivity disorder and emotional dysregulaton. Int J Neuropsychopharmacol.

[145] Cimino S, Carola V, Cerniglia L (2020). The μ-opioid receptor gene A118G polymorphism is associated with insecure attachment in children with disruptive mood regulation disorder and their mothers. Brain Behav.

[146] Haller SP, Kircanski K, Stringaris A (2020). The clinician affective reactivity index: validity and reliability of a clinician-rated assessment of irritability. Behav Ther.

[147] Haller SP, Stoddard J, Pagliaccio D (2020). Computational modeling of attentional impairments in disruptive mood dysregulation and attention deficit/hyperactivity disorder. Biol Psychiat.

[148] Johnstone JM, Leung BMY, Srikanth P (2020). Development of a composite primary outcome score for children with attention-deficit/hyperactivity disorder and emotional dysregulation. J Child Adolesc Psychopharmacol.

[149] Le JF, Feygin Y, Creel L (2020). Trends in diagnosis of bipolar and disruptive mood dysregulation disorders in children and youth. J Affect Disord.

[150] Tseng W-L, Scheinost D, Pine DS (2020). Functional connectivity during frustration is predictive of irritability in youth. Biol Psychiat.

