# Supplementary material for: Diagnostic instruments for the assessment of disruptive mood dysregulation disorder: a systematic review of the literature
Source: Eur Child Adolesc Psychiatry. 2021 Jul 7;32(1):17–39. doi: 10.1007/s00787-021-01840-4 (PMC9908712; doi:10.1007/s00787-021-01840-4)
Supplement: Supplementary file 1 — Supplementary file1 (DOCX 13 kb) [file 787_2021_1840_MOESM1_ESM.docx]

# **Supplementary material**

**Diagnostic criteria for DMDD according to the DSM-5**

A. Severe recurrent temper outbursts manifested verbally (e.g., verbal rages) and/or behaviorally (e.g., physical aggression toward people or property) that are grossly out of proportion in intensity or duration to the situation or provocation.

B. The temper outbursts are inconsistent with developmental level.

C. The temper outbursts occur, on average, three or more times per week.

D. The mood between temper outbursts in persistently irritable or angry most of the day, nearly every day, and is observable by others (e.g., parents, teachers, peers).

E. Criteria A–D have been present for 12 or more months. Throughout that time, the individual has not had a period lasting 3 or more consecutive months without all of the symptoms in Criteria A–D.

F. Criteria A and D are present in at least two of the three settings (i.e., at home, at school, with peers) and are severe in at least one of these.

G. The diagnosis should not be made for the first time before age 6 years or after age 18 years.

H. By history or observation, the age of onset of Criteria A-E is before 10 years.

I. There has never been a distinct period lasting more than 1 day during which the full symptom criteria, except duration, for a manic or hypomanic episode have been met.

Note: Developmentally appropriate mood elevation, such as occurs in the context of a highly positive event or its anticipation, should not be considered as a symptom of mania or hypomania.

J. The behaviors do not occur exclusively during an episode of major depressive disorder and are not better explained by another mental disorder (e.g., autism spectrum disorder, posttraumatic stress disorder, separation anxiety disorder, persistent depressive disorder [dysthymia]).

Note: This diagnosis cannot coexist with oppositional defiant disorder, intermittent explosive disorder, or bipolar disorder, though it can coexist with others, including major depressive disorder, attention-deficit/hyperactivity disorder, conduct disorder, and substance use disorders. Individuals whose symptoms meet criteria for both disruptive mood dysregulation disorder and oppositional defiant disorder should only be given the diagnosis of disruptive mood dysregulation disorder. If an individual has ever experienced a manic or hypomanic episode, the diagnosis of disruptive mood dysregulation disorder should not be assigned.

K. The symptoms are not attributable to the physiological effects of a substance or to another medical or neurological condition.
